# Measurement properties of the Iranian version of the breast cancer perception scale (BCPS) according to the COSMIN checklist

**DOI:** 10.1186/s12885-024-12493-2

**Published:** 2024-06-18

**Authors:** Sepideh Mashayekh-Amiri, Mohammad Asghari Jafarabadi, Mina Hosseinzadeh, Elham seyed Kanani, Mojgan Mirghafourvand

**Affiliations:** 1 Students Research Committee, Midwifery Department, Faculty of Nursing and Midwifery, Tabriz University of Medical sciences, Tabriz, Iran; 2 Cabrini Research, Cabrini Health, Melbourne, VIC 3144 Australia; 3 School of Public Health and Preventative Medicine, Faculty of Medicine, Nursing and Health Sciences, Monash University, VIC 3800, Melbourne, Australia; 4 Road Traffic Injury Research Center, Tabriz University of Medical Sciences, Tabriz, Iran; 5 Department of Community Health Nursing, Nursing and Midwifery Faculty, Tabriz University of Medical Sciences, Tabriz, Iran; 6 Department of Community Health Nursing, Faculty of Nursing and Midwifery, Tabriz University of Medical sciences, Tabriz, Iran; 7 Social Determinants of Health Research Center, Department of Midwifery, Faculty of Nursing and Midwifery, Tabriz University of Medical Sciences, Tabriz, Iran; 8 Medical Philosophy and History Reseach Center, Tabriz University of Medical Sciences, Tabriz, Iran

**Keywords:** Breast cancer, Perception, Psychometric, Patient-reported outcome (PRO), COSMIN checklist, Reliability, Iranian validation

## Abstract

**Background:**

Breast cancer is a prevalent cancer characterized by its aggressive nature and potential to cause mortality among women. The rising mortality rates and women’s inadequate perception of the disease’s severity in developing countries highlight the importance of screening using conventional methods and reliable scales. Since the validity and reliability of the breast cancer perception scale (BCPS) have not been established in the Iranian context. Therefore, this study aimed to determine the measurement properties of the BCPS in women residing in Tabriz, Iran.

**Methods:**

The present study comprised a cross-sectional design, encompassing a sample of 372 Iranian women. The participants were selected through a multi-stage cluster random sampling technique conducted over a period spanning from November 2022 to February 2023. The measurement properties of the Iranian version of BCPS were assessed following the guidelines outlined in the COSMIN checklist. This involved conducting various steps, including the translation process, reliability testing (internal consistency, test-retest reliability, and measurement error), and methodological tests for validity (content validity, face validity, construct validity, and hypothesis testing). The study also investigated the factors of responsiveness and interpretability. The presence of floor and ceiling effects was assessed.

**Results:**

The internal consistency of the scale was assessed using Cronbach’s alpha, yielding a satisfactory value of 0.68. Additionally, McDonald’s omega (95% CI) was computed, resulting in a value of 0.70 (0.66 to 0.74). Furthermore, the test-retest reliability was evaluated, revealing a high intraclass correlation coefficient (ICC) of 0.97 (95% CI: 0.94 to 0.99). The CVI, CVR, and impact scores of the BCPS were determined to be 0.98, 0.95, and 3.70, respectively, indicating favorable levels of content and face validity. To assess construct validity, an examination of the Exploratory Factor Analysis (EFA) was conducted on a set of 24 items. This analysis revealed the presence of six distinct factors, which collectively accounted for 52% of the cumulative variance. The fit indices of the validity model (CFI = 0.91, NFI = 0.96, RFI = 0.94, TLI = 0.90, χ^2^/df = 2.03, RMSEA = 0.055 and SRMR = 0.055) were confirmed during the confirmatory factor analysis (CFA). The overall score of BCPS exhibited a ceiling effect of 0.3%. The floor effect observed in the overall score (BCPS) was found to be 0.5%. Concerning the validation of the hypothesis, Spearman’s correlation coefficient of 0.55 was obtained between the BCPS and the QLICP-BR V2.0. This correlation value signifies a statistically significant association. Furthermore, it is worth noting that the minimum important change (MIC) of 3.92 exhibited a higher value compared to the smallest detectable change (SDC) of 3.70, thus suggesting a satisfactory level of response.

**Conclusions:**

The obtained findings suggest that the Iranian version of the BCPS demonstrates satisfactory psychometric properties for assessing the perception of breast cancer among Iranian women. Furthermore, it exhibits favorable responsiveness to clinical variations. Consequently, it can serve as a screening instrument for healthcare professionals to comprehend breast cancer and as a reliable tool in research endeavors.

## Background

Breast cancer is a significant global health issue [1], accounting for approximately 30% of cancer cases among women [2]. It is recognized as the second-leading cause of mortality in developed nations and the third-leading cause of mortality in less developed nations [3]. Based on the findings of the Global Cancer Incidence, Mortality and Prevalence (GLOBOCAN) report in 2020, it was determined that there were an estimated 2,261,419 million new cases (1 in 4 new cancer cases) (11.7%), and 684,996 (1 in 6 deaths) (6.9%) fatalities [4]. It is also predicted that these figures will reach 2,964,197 in 2040 (31% rise from 2018) [5], and 4.4 million in 2070 (110% rise from 2018) [6]. According to the report of the American Cancer Society in 2024, the number of new cases of female breast cancer in the United States was 310,720 and the number of deaths was 42,250 [7]. Approximately two-thirds of these fatalities are documented in regions with lower levels of development [4]. Alternatively, based on the projection provided by the World Health Organization (WHO), it is anticipated that by 2050, approximately 2.3 million women will receive a diagnosis of breast cancer [8]. Breast cancer is recognized as a highly costly form of cancer on a global scale, with an estimated annual expenditure of approximately 88 billion dollars. Failing to promptly diagnose and conduct screening examinations, coupled with the consequential impact on the entire family unit, imposes substantial financial burdens on society [9].

Breast cancer exhibits the highest prevalence and mortality rates among women in the Eastern Mediterranean region (EMR), encompassing Iran when compared to other forms of cancer. Breast cancer is widely recognized as the predominant form of cancer in Iran, ranking as the fifth-highest cause of mortality among women in the country [10]. The Age-standardized rate (ASR) incidence rate is approximately 28 per 100,000 individuals, exhibiting a recent upward trend [11]. Based on a systematic review, it has been documented that the incidence rate of breast cancer in Iran stands at 23.6% [12]. The reported prevalence of this cancer in the United States (US) is approximately 13%, indicating that one out of every eight individuals is affected [13].

Breast cancer is correlated with numerous risk factors, a significant proportion of which remain unidentified. The findings of a systematic review conducted in 2020 in Iran reveal various risk factors associated with breast cancer. These factors include family history, hormone replacement therapy (HRT), exposure to passive smoking, advanced maternal age during pregnancy, history of abortion, consumption of sweets, and possession of the Arg/Arg genotype. These factors have been found to potentially elevate the risk of developing breast cancer. Conversely, certain factors such as the late onset of menstruation, nulliparity, breastfeeding for a duration of 13 to 24 months, regular physical exercise, and consumption of vegetables have been observed to have a protective effect against the incidence of breast cancer [14].

It is noteworthy that the incidence of breast cancer among Iranian women occurs at an age approximately 10 years earlier compared to women in other developed nations. According to various studies, there has been a documented increase in the prevalence of breast cancer among women under the age of 40 in recent years [15]. The rise in its occurrence in developing nations is primarily attributed to alterations in lifestyle and reproductive behaviors [16]. The majority of female individuals afflicted with breast cancer receive a diagnosis during the later stages of the ailment, thereby correlating with an elevated mortality rate. Hence, it has been observed that early detection of breast cancer leads to a significant improvement in both survival rates and treatment outcomes, with a reported increase of 90% [17].

The United States Office of Disease Prevention and Health Promotion’s Healthy People 2020 initiative has as its goals the improvement of breast cancer diagnostic procedures for women, a decrease in the prevalence of cases of end-stage cancer, and a reduction in breast cancer mortality rates. Conversely, in the case of cancers that exhibit both genetic and environmental risk factors, it is imperative to adopt strategies that prioritize modifiable risk factors and early detection. Hence, the implementation of a preventive strategy aimed at early detection, which incorporates the evaluation of knowledge on breast cancer and its associated risks, assumes paramount significance [18].

The perception of breast cancer is a crucial subjective psychological phenomenon that is associated with the evaluation of potential threats. This evaluation is linked to an individual’s assessment of their susceptibility to the disease and the probability of gaining advantages from engaging in preventive measures [15]. Various studies have indicated that risk perception is a significant determinant of preventive health-related behaviors, such as screening. The motivation to undergo screening tests can be influenced by individuals’ perceptions of the risk associated with breast cancer [16]. According to the literature, screening tests play a crucial role in mitigating complications and mortality associated with breast cancer [17].

Various studies have documented divergent findings regarding the correlation between the perception of breast cancer risk and the utilization of screening tests, such as mammography [18]. Research findings indicate that the implementation of mammography screening during the age range of 40 to 49 years has been associated with a reduction in mortality rates of approximately 15 to 20% [19].

According to a study conducted on families to assess their perception of breast cancer risk, the rate of adherence to screening tests in Germany was found to be 83% [20]. Conversely, a research study conducted in Iran examined the adherence rate of women aged 35 to 69 years to mammography, as recommended by screening programs. The findings revealed that in urban areas, the adherence rate was 8.3%, while in rural areas, it was 3.16% [1]. Therefore, the perception level that women possess regarding breast cancer has the potential to influence their subsequent actions, such as seeking medical evaluation and undergoing screening procedures like breast self-examination (BSE), clinical breast examination (CBE), and mammography. Therefore, it is imperative to assess the perception of breast cancer in women using a multidimensional approach [20].

Numerous methodologies have been suggested for assessing the perception of breast cancer risk, which can be categorized into two distinct types: evaluation of the objective perception of risk (i.e., actual risk) and evaluation of the subjective perception of risk [21]. Currently, Gill’s model predominantly serves as a tool for conducting quantitative risk assessments. This approach aims to objectively evaluate the actual risk by considering the attributes of risk factors [22]. The second method entails the assessment of self-perceived risk, which can be anticipated by gauging individuals’ mental perceptions using a visual analog scale (VAS). Despite the presence of a multitude of tools within this domain, their practicality appears to be limited as they do not provide comprehensive coverage of all the factors that influence behaviors related to the diagnosis of breast cancer [23].

Taylan et al. (2021) developed the BCPS in Turkey, considering the health belief model for the first time. This scale encompasses various domains, including Perceived knowledge, Perceived treatment belief, Perceived need for health check, Perceived stigma, Perceived fear and Perceived risk. The utilization of this scale offers several benefits in assessing women’s perceptions regarding the factors influencing breast cancer diagnostic behavior comprehensively. Furthermore, it is worth noting that the extent of women’s perceived knowledge of breast cancer has not been quantitatively evaluated thus far. Consequently, this tool serves as a distinctive scale specifically designed to measure women’s knowledge regarding cancer. Additionally, it quantifies the dimensions of the breast [23].

The Health Belief Model (HBM) was initially formulated by Becker et al., in 1974 to comprehend health-related protective behaviors [24]. The evaluation of perceived risk, employing the HBM, has been validated in various studies examining screening behaviors, such as those related to breast cancer diagnostics [25–27]. The model encompasses various dimensions, namely perceived sensitivity, perceived severity, perceived benefits, perceived barriers, self-efficacy, and guidance for action. Based on the presented model, individuals’ healthcare behaviors can be subject to influence from factors such as perception, beliefs, values, and attitudes. By identifying and modifying an individual’s perceptions, beliefs, and attitudes, the effectiveness of healthcare education or treatment can be enhanced [26].

However, it is crucial to assess the methodological rigor of studies that evaluate the measurement properties of instruments used to measure health-related patient-reported outcomes (HR-PROs) [28]. The Consensus-Based Standards for the Selection of Health Status Measurement Instruments (COSMIN) checklist was developed by Mokkink et al. in 2010 through a consensus-based approach utilizing the Delphi method [28]. The COSMIN list is widely regarded as a highly comprehensive set of criteria for selecting an appropriate tool. It serves as a valuable guide for researchers, offering a range of logical indicators that aid in the process of tool selection [29].

Given the rising incidence of breast cancer, the significance of early screening, and the potential influence of risk perception on women’s adoption of preventive behaviors, such as breast screening methods, it is pertinent to assess the level of knowledge regarding breast cancer when devising interventions aimed at modifying health behaviors. It is worth noting that the validity and reliability of the aforementioned assessment tool have not been established in Iran. The present study was undertaken to conduct a measurement propertice of the BCPS in women residing in Tabriz city-Iran, by using the COSMIN checklist.

## Methods

### Study aim

The present study was conducted with the aim of determining the measurement properties of the breast cancer risk perception scale (BCPS) in according to COSMIN checklist in women in Tabriz, Iran.

### Validity procedure

The measurement properties of the Iranian version of BCPS were assessed following the guidelines outlined in the COSMIN checklist [29]. This involved conducting various steps, including the translation process, reliability testing (internal consistency, test-retest reliability, and measurement error), and methodological tests for validity (content validity, face validity, construct validity, and hypothesis testing). The study also investigated the factors of responsiveness and interpretability. The presence of floor and ceiling effects was assessed.

### Translation process

Initially, permission to use the BCPS was obtained by sending an email from the original designer of the instrument (Taylan et al.) [23]. Efforts were made to maintain the integrity of the original intent during the translation process. Following the recommendations made by the WHO, EORTC Quality of Life Group Translation Procedure Guidelines and expert panel review, this was performed [30]. The translation process involves the utilization of two distinct methods. The two methods utilized in this study are the Forward-Backward method (FB) and the Dual Panel method (DP), which were implemented throughout four distinct stages. The process consisted of four stages: forward translation, backward translation, pre-testing and cognitive interviewing, and the final version.

During the initial phase of translation, the original English version of the instrument was administered to two individuals who were native Persian speakers, proficient in English, and possessed expertise in the development of the instrument as well as knowledge in the field of breast cancer. Translators were subsequently instructed to translate the tool in a fully autonomous and individual manner, with a focus on conceptual rather than literal translations. Additionally, they were encouraged to use language that would be comprehensible to the majority of the target audience. Ultimately, two translators looked into the discrepancies between the two translated versions, which led to a reconciled translation. Subsequently, the identified issues were addressed, leading to the presentation of a unified version [31]. Subsequently, the backward translation method was employed to guarantee a comprehensive correspondence between the Persian translation and the original version. The translated questionnaire from the preceding stage was administered to two individuals who are native English speakers. These individuals were not involved in the forward translation process and had no prior exposure to the original version of the questionnaire. They were instructed to retranslate the questionnaire back into English. The concluding report at the culmination of this phase encompassed the following components: two forward translations from the English language to Farsi, a reconciled translation, two backward translations from Farsi to English, and the incorporation of any supplementary remarks regarding the translations provided by the panel of experts. Ultimately, before implementing the tool in the intended population, it is imperative to conduct a pilot study. To achieve the intended objective, a questionnaire was administered to a sample of ten qualified female participants. Based on the feedback received from these participants regarding the ease of completing the instrument, grammar, comprehensibility, and writing style, modifications were made to the Persian version, and the revised version was finalized and presented [31].

### Validation study

This cross-sectional study aimed to assess the measurement properties of the Iranian version of the BCPS among a sample of 372 Iranian women who sought healthcare services at Tabriz health centers affiliated with Tabriz University of Medical Sciences in two separate secondary samples (172 participants via exploratory factor analysis and 200 participants via confirmatory factor analysis). The study was conducted following the approval of the Ethics Committee of Tabriz University of Medical Sciences (ref: IR.TBZMED.REC.1401.390) from November 2022 to February 2023. It is important to acknowledge that informed consent was obtained from all participants. The study was conducted in adherence to the applicable regulations of the Ethics Committee of the University of Medical Sciences and the Declaration of Helsinki.

Among the 410 women, it was found that 16 participants did not satisfy the predetermined eligibility criteria, leading to their exclusion from the research investigation. Out of the remaining 394 participants who met the eligibility criteria, a total of 22 participants expressed their unwillingness to participate in the study. Ultimately, a total of 372 participants were incorporated into the research investigation. The response rate for the study was 94%, (372/394). In the sampling procedure, the cluster method was employed to randomly select a quarter of the 92 health centers in Tabriz City. The selection process was facilitated through the utilization of the website (www.random.org). In addition to their contact information, which was obtained from the SIB system (integrated health system), women were selected at random from the compiled list. To clarify, the selection of women from each center was determined based on the proportional sampling method, and the process of randomly selecting women was carried out utilizing the aforementioned website. Following a telephone conversation with the participants, wherein the researcher offered a concise overview of the research, the researcher proceeded to invite the women to attend the designated health center within the specified timeframe. The purpose of this visit was to provide additional explanations and administer the questionnaires. Upon conducting a visit and assessing the fundamental aspects such as basic information and inclusion and exclusion criteria, the individual proceeded to furnish the concerned parties with extensive details related to the research, its advantages, outcomes, and the confidentiality of the data. It is important to acknowledge that the random selection of participants was conducted before the assessment of their eligibility criteria. Following their visit to the health center, participants underwent a comprehensive evaluation to gather baseline data and determine their eligibility. Full information regarding the research objectives and methodology was exclusively provided to individuals who satisfied the predetermined eligibility criteria, and only they were extended an invitation to participatein the study. After agreeing to participate in the study, the participants proceeded to complete the informed consent form, the questionnaire of socio-demographic characteristics, and the BCPS.

The study’s inclusion criteria encompassed women who were at least 20 years old, exhibited no indications of abnormal breast lesions during clinical examination, and possessed the necessary literacy skills to complete the questionnaire. The study excluded individuals who met the following criteria: a confirmed diagnosis of breast cancer as documented in medical records, a history of cosmetic breast surgery, and impairment in communication skills related to hearing and speaking, and an inability to physically, cognitively, or mentally respond to questions.

### Measures

#### Socio-demographic questionnaire

The questionnaire included questions regarding socio-demographic factors such as age, spouse age, marital status, educational level, job, income, breast cancer history, history of hormone therapy, family history of breast cancer and menopause status.

### Breast cancer perception scale

Taylan et al. (2021) in Turkey [23] developed the BCPS. The present tool is grounded in the theoretical framework of the HBM and comprises a set of 24 items designed to assess women’s perceptions of breast cancer. The construct comprises six sub-dimensions, including Perceived knowledge, Perceived treatment belief, Perceived need for health check, Perceived stigma, Perceived fear and Perceived risk, which are assessed using a five-point Likert scale. The responses span a spectrum from strongly disagree (1) to strongly agree (5). The scale’s validity and reliability have been empirically established within the specific demographic of Turkish women in 2021. The lower bound of the scoring range for this questionnaire is 24, while the upper bound is set at 120. A positive correlation exists between higher scores and a greater level of women’s perception of breast cancer [23].

### Sample size determination

It is imperative to ascertain the appropriate sample size to conduct the factor analysis procedure. According to a rule of thumb, the classification of sample size for EFA is as follows: a sample size of 50 is considered very poor, 100 is poor, 200 is fair, 300 is good, 500 is very good, and 1000 is excellent [32]. To ensure the reliability and validity of the results, it is necessary to have an appropriate sample size when conducting factor analysis. This study incorporated the guidelines proposed by Nunnally [33], which recommend a sample size of 5 to 10 samples for each instrument question to facilitate the generalizability of the findings to the broader community. Under these guidelines, it was deemed appropriate to utilize a sample size of 10 samples for each case, taking into account that the tool consisted of 24 items. Therefore, initially, a minimum of 240 samples were considered necessary. Nevertheless, it is imperative to take into account the effectof the cluster sampling technique employed in the research. The cluster sampling method introduces a factor of intra-cluster correlation that necessitates its inclusion in the calculation of the sample size [33]. To address this issue, a design effect of 1.5 was employed to modify the sample size. Due to a 10% attrition rate, the sample size has consequently expanded to encompass a total of 372 participants.

### Statistical analysis

The statistical analysis was conducted using the SPSS software package (version 16, IBM Corp., Armonk, NY, USA), STATA14 (Statcorp, College Station, Texas, USA), and R software 4.2 (Psych package). To examine socio-demographic data, descriptive statistics were employed, including frequency (percentage) for qualitative variables, minimum and maximum values, and mean ± standard deviation (SD) for quantitative variables. The evaluation encompassed methodological testing, which involved the assessment of reliability, validity, and responsiveness. Exploratory factor analysis (EFA) and confirmatory factor analysis (CFA) techniques were employed to assess construct validity on a larger scale. The direct oblimin method was employed in the EFA. Bartlett’s test for sphericity and KMO’s test for assessing the adequacy of scale content and sample size were conducted. The CFA methodology was employed to assess the factor structure and factor loadings of the scale. In conclusion, an assessment was conducted to evaluate the reliability of the study, specifically focusing on internal consistency, test-retest reliability, and measurement error. Finally, the presence of ceiling and floor effects was assessed.

### Methodological testing according to the COSMIN checklist

#### Reliability

Reliability refers to the extent to which a measurement is devoid of any errors that may arise during the measurement process. The evaluation of reliability primarily involves the assessment of three key characteristics: internal consistency, test-retest reliability, and measurement error [29].

### Internal consistency

Internal consistency refers to the extent of interconnectedness among items. It serves as an estimation of the correlation level between the variables that constitute the intended structure or instrument [29]. The internal consistency of the instrument as a whole and its six subscales was assessed using both Cronbach’s alpha coefficient and McDonald’s omega coefficient. A minimum threshold of 0.7 was deemed necessary for both Cronbach’s alpha and MacDonald’s Omega coefficients to establish satisfactory internal consistency [34].

### Test–retest reliability

Test-retest reliability refers to the extent to which the outcomes of a patient with identical health conditions remain consistent over a while [29]. Following the guidelines outlined in the COSMIN manual, a test-retest procedure was conducted with a minimum interval of two weeks. This time frame was chosen to prevent participants from recalling their previous responses and to account for any potential changes in their health status [29]. To achieve the intended objective, a survey was administered to a cohort of 30 female participants on two separate occasions, with a 14-day gap between each administration. The resulting scores were subsequently utilized to assess the reliability of the survey instrument through the application of the intraclass correlation coeficient (ICC). A reliability coefficient greater than 0.7 was deemed advantageous [34].

### Measurement error

Measurement error is considered one of the key indicators of measurement and test reliability. In essence, it refers to the presence of both systematic and random errors in the patient’s score, which cannot be attributed to genuine variations in the construct under consideration. The calculation of the standard error of measurement (SEM) involves the use of the formula (SEM = SD√1-ICC), where SD represents the standard deviation [34]. The concept of the smallest detectable change (SDC) pertains to the minimum magnitude of an individual score change that can be accurately interpreted as a genuine change. The calculation of the SDC is determined by employing the formula (SDC = SEM*1.96*√2). A reduced level of the SDC corresponds to an increased level of measurement sensitivity [34].

### Validity

Validity refers to the extent to which a given instrument accurately measures the specific characteristic it is designed to assess [29].

### Face validity

Face validity is a concept that pertains to the extent to which the items within an instrument, specifically the HR-PRO, accurately represent the underlying construct that is intended to be measured [29]. The researchers conducted an assessment of face validity using both qualitative and quantitative methods. To conduct a qualitative assessment of face validity, a sample of 10 women from health centers in Tabriz City was selected using a convenience sampling method. This sample then examined the initial questionnaire. The participants assessed the quality, level of difficulty, lack of relevance, and degree of ambiguity of the items. To evaluate the face validity, item impact scores were quantitatively computed. During this phase, the aforementioned participants assessed each item on a 5-point Likert scale, ranging from “completely important” to “not at all important,” with scores ranging from 5 to 1 (representing “completely important,” “important,” “moderately important,” “slightly important,” and “not important,” respectively). The impact score is calculated by multiplying the Frequency (expressed as a percentage) by the Importance (Impact Score = Frequency (%) × Importance). Items with an impact score exceeding 1.5 were deemed appropriate and were subsequently retained for further stages of analysis [35].

### Content validity

The degree to which the content of an HR-PRO instrument effectively represents the construct that is intended to be assessed [29]. Both qualitative and quantitative methods were used to examine the validity of the content of the questionnaire. To assess the credibility of the qualitative content, a group of ten experts, including three experts in reproductive health, two specialists in midwifery, three specialists in medical-surgical nursing, and two specialists in community health nursing, were invited to provide their insights and opinions on topics considering grammar, vocabulary choice, item arrangement, and scoring.

The inclusion criteria of the experts to determine content validity include voluntary participation, faculty members with the rank of assistant professor and above, PhDs of midwifery/nursing and individuals with clinical experience in breast cancer. The process of assessing quantitative content validity involves the calculation of two measures: the content validity ratio (CVR) and the content validity index (CVI) [36]. To fulfill the objective, a questionnaire comprising questions organized into two overarching categories was distributed to each expert. In the initial phase, the participants assessed the items using a 3-point Likert scale (necessary, useful but not necessary, not necessary) to ascertain the CVR, which was computed using the following mathematical expression:

CVR= (Ne-N/2)/ (N/2).

Where, “Ne” represents the count of experts who have chosen the “necessary” option, and N denotes the total number of experts. Regarding Lawshe table, a CVR > 0.62 for a sample size of 10 individuals, confirms the essentiality of the items under investigation [37].

Subsequently, the CVI review underwent evaluation by an identical group of 10 experts. Concerning this matter, questions have been raised regarding the three criteria of relevance, clarity, and simplicity for each item. These criteria have been assessed using a four-point Likert scale, which includes options such as irrelevant, somewhat relevant, relevant, and completely relevant. The assessment is based on the content validity index [38] developed by Waltz and Basel. The level of relevance, clarity, and simplicity was assessed by experts based on their subjective evaluation, and then the CVI was computed using the following formula:

CVI = number of experts giving a rating of 3 and 4 / total number of experts.

CVIs higher than 0.79, between 0.70 and 0.79, and less than 0.70 were considered acceptable, in need of correction, and unacceptable, respectively [39].

### Construct validity

The concept of construct validity pertains to the extent to which the scores obtained from an HR-PRO instrument align with the anticipated hypotheses. This alignment can be observed in terms of internal relationships, relationships with scores obtained from other instruments, or differences between relevant groups. This assessment is contingent upon the assumption that the HR-PRO instrument possesses validity. The concept of validity pertains to the extent to which a given measure accurately assesses the construct it is intended to measure. The three aspects encompassed in this study are as follows: structural validity, which pertains to the internal relationships within the construct; hypothesis testing; and cross-cultural validity, which focuses on the relationships with scores on other instruments or differences between relevant groups [29].

### Structural validity

The suitability of the data for EFA was assessed by employing the Kaiser-Meyer Olkin (KMO) criterion and Bartlett’s test of sphericity. The KMO test is a statistical measure that quantifies the proportion of variance in the questions that can be attributed to the primary factors. Typically, values falling within the range of 0.8–1 are indicative of adequate data sampling to conduct factor analysis. However, when the value of the statistic falls below 0.7, it indicates that the sample size is insufficient, necessitating the implementation of corrective actions [40].

Bartlett’s test of sphericity is a frequently employed statistical test to assess the appropriateness of data for factor analysis. The significance of this test serves as an indicator of the suitability of the data for factor analysis [40]. The process of extracting factors from the 24 items of the questionnaire was conducted using the principal component analysis method, employing varimax rotation (direct oblimin). The determination of the number of factors was based on the criterion of an Eigenvalue greater than 1 and the examination of the Scree plot. In this analysis, a minimum factor loading threshold of 0.3 was utilized for the extraction of factors. In contrast, CFA employs the maximum likelihood method to estimate the model’s fit indices, and a range of indices are utilized to assess the appropriateness of the model. This study assessed the adequacy of the model by employing the indicators outlined below [41]:

Root mean score error of approximation (RMSEA < 0.08), standardized root mean square residual (SRMR < 0.10), normed Chi^2^ (x^2^ / df) < 5, comparative fit indices including comparative fit index (CFI > 0.90), Bentler-Bonett Normed Fit Index (NFI) > 0.90, Relative fit index (RFI) > 0.90 and Tucker-Lewis Index (TLI) > 0.90.

### Hypothesis testing

The process of hypothesis testing is characterized by its continuous and iterative nature. Hypotheses serve as a means to express the anticipated direction and magnitude of correlations or differences between the construct under investigation and other constructs. As the number of hypotheses tested regarding the alignment between the data and pre-existing hypotheses increases, a greater amount of evidence supporting construct validity is accumulated [29]. To assess construct validity, an analysis of the hypotheses that were previously formulated was conducted. In this study, it was postulated that the BCPS would exhibit a strong correlation with other subjective scales, such as quality-of-life instruments for cancer patients (QLICP-BR V2.0). Hence, confirmation of the desired hypothesis can be achieved when the Pearson correlation coefficient exceeds 0.5. Furthermore, the study computed the floor and ceiling effect (F/C) as well as the proportion of women who achieved the minimum and maximum scores. F/C effects refer to the percentage of individuals who achieve the highest (ceiling) or lowest (floor) possible scores within a specific domain. These effects serve as indicators of a questionnaire’s sensitivity and coverage at the extreme ends of the scale. In the context of this study, a problematic scenario is defined as a situation where 15% or more of the respondents fall into either the ceiling or floor category [42].

#### Responsiveness

Measurement instruments should possess a high degree of sensitivity to detect and accurately capture changes, while also demonstrating a responsive nature to promptly reflect these changes. According to the COSMIN checklist, responsiveness refers to the capacity of an HR-PRO instrument to accurately identify alterations in the construct being assessed over a while [29]. Terwee et al. [34] argue that responsiveness can be assessed by examining the relationship between the smallest detectable change (SDC) and the minimally important change (MIC). If the value of SDC is less than MIC, then the responsiveness is confirmed.

### Interpretability

Interpretability refers to the extent of qualitative significance, specifically the minimally important changes (MIC) within the instrument. The extent to which quantitative instrument scores or changes in scores can be attributed to qualitative meaning, such as clinical or commonly understood meanings, has been discussed [29]. The estimation of the minimum important change (MIC) was conducted by dividing the standard deviation (SD) by two, as outlined in the study conducted by Norman et al. [43].

## Results

### Descriptive characteristics of participants

This study involved the participation of 372 women. The participants were randomly split into two groups, one group of 172 participants for EFA and another group of 200 participants for CFA. The average age was 52.7 and 52.3 years with a standard deviation of 9.5 and 8.5 years in EFA and CFA group, respectively. A significant majority of the individuals surveyed were married (78.5%, 80.5% in EFA and CFA group, respectively), and 73.8%, 61.0% of them identified themselves as housewives in EFA and CFA group, respectively. Table 1 provides a summary of the additional socio-demographic characteristics of the two groups of participants.

**Table 1:** Socio-demographic characteristics of participants for EFA and CFA (*n* = 372)

Characteristics	EFA (*n* = 172)		CFA (*n* = 200)	
	Mean	SD	Mean	SD
**Age** (Year)	52.7	9.5	52.3	8.5
**Spouse Age** (Year)	56.8	9.1	56.6	9.0
**Number of children**	2.5	1.6	2.1	1.1
	**Number**	**Percent**	**Number**	**Percent**
**Marital status**				
Single	37	21.5	39	19.5
Married	135	78.5	161	80.5
**Number of children**				
**3>**	101	58.7	139	69.5
**3≤**	57	33.1	43	21.5
**Education level**				
Intermediate or below	119	69.2	100	50.0
Diploma and university	53	30.8	100	50.0
**Job**				
Housewife	127	73.8	122	61.0
Employee	45	26.2	78	39.0
**Income**				
Not at all sufficient	82	47.7	64	32.0
Relatively sufficient	64	37.2	105	52.5
Completely sufficient	26	15.1	31	15.5
**Breast cancer history**				
Yes	12	7.0	37	18.5
No	160	93.0	163	81.5
**History of hormone therapy**				
Yes	44	25.6	44	22.0
No	128	74.4	156	78.0
**Family history of breast cancer**				
Yes	32	18.6	42	21.0
No	140	81.4	158	79.0
**Menopause**				
Yes	93	54.1	112	56.0
No	79	45.9	88	44.0

^*^Standard deviation

In the present study, the mean (SD) of the entire BCPS scale was 61.66 (8.44), with a range of obtainable scores from 24 to 120. The mean (SD) for the six extracted factors, namely Perceived fear, Perceived knowledge, Perceived treatment belief, Perceived risk, Perceived need for a health check, and Perceived stigma, were respectively 8.28 (4.02), 11.63 (3.79), 10.5 (2.41), 9.37 (2.12), 10.19 (3.22), and 12.15 (2.65).

#### Reliability

The values of Cronbach’s alpha and McDonald’s omega (95% CI) were found to be 0.68 and 0.70 (0.66 to 0.74), respectively. These results suggest that the questionnaire exhibits satisfactory internal consistency. Also, the ICC (95% CI) gave a value of 0.97 (0.94 to 0.99). Standard error of measurment is a statistical metric utilized to assess the accuracy and consistency of a given measurement. The SEM value in this study was determined to be 1.36. This implies that upon conducting multiple iterations of the measurement, it is anticipated that the recorded values will fall within a range of ± 1.36 units with the actual score. Moreover, the SDC denotes the smallest detectable change that can be consistently detected by the measuring apparatus. Within the given framework, the value of SDC was ascertained to be 3.73 units. This implies that any deviation in the measured quantity that is less than 3.73 units may not be discernible due to measurement errors and can be regarded as insignificant (Table 2).

**Table 2:** Scale subscale scores, Stability Coefficients, Interclass Correlation Coefficient of the BCPS (*n* = 372)

Factors	Cronbach’s α coefficient	McDonald’s omega (95% CI)	ICC (95% CI)	SEM	SDC	MIC	AVE	Floor effect (%)	Ceiling effect (%)
Perceived fear	0.94	0.94 (0.93, 0.95)	0.89 (0.78, 0.95)	0.91	2.50	1.37	0.722	26.9	2.2
Perceived knowledge	0.88	0.88 (0.86, 0.90)	0.96 (0.91, 0.98)	0.79	2.17	1.97	0.533	3.5	4.0
Perceived treatment belief	0.77	0.77 (0.73, 0.81)	0.95 (0.89, 0.98)	0.57	1.56	1.27	0.549	3.2	0.3
Perceived risk	0.83	0.60 (0.54, 0.66)	0.99 (0.98, 0.99)	0.19	0.52	0.94	0.548	1.3	1.1
Perceived need for health check	0.64	0.66 (0.61, 0.71)	0.97 (0.94, 0.99)	0.58	1.59	1.68	0.548	4.6	0.3
Perceived stigma	0.64	0.66 (0.60, 0.71)	0.98 (0.96, 0.99)	0.41	1.13	1.44	0.545	0.8	0.8
Total	0.68	0.70 (0.66, 0.75)	0.97 (0.94, 0.99)	1.36	3.73	3.92	0.574	0.5	0.3

SD standard deviation; CI confidence interval; ICC intra class correlation coefficient; SEM Standard error of the measurement; SDC smallest detectable change detectable change; MIC minimal important change; AVE average variance extracted (acceptable if AVE > 0.5, the threshold is 0.36–0.5); BCPS breast cancer perception scale

### Validity

The tool’s content and face validity were assessed using the CVI (CVI range: 0.87–1.00), CVR (CVR range: 0.75–1.00), and impact scores (3.06–4.00), which yielded values of 0.98, 0.95, and 3.70, respectively.

The construct validity investigation involved conducting an EFA on a set of 24 items. The resulting Kaiser-Meyer-Olkin (KMO) value of 0.71 was obtained at a statistically significant level of less than 0.001, indicating that the sample size in the current study was sufficient. Furthermore, the statistical analysis revealed that Bartlett’s test of sphericity yielded a significant result (*p* ≤ 0.001), indicating that factor analysis was appropriately conducted based on the correlation matrix in the sample under investigation.

**Fig. 1:**
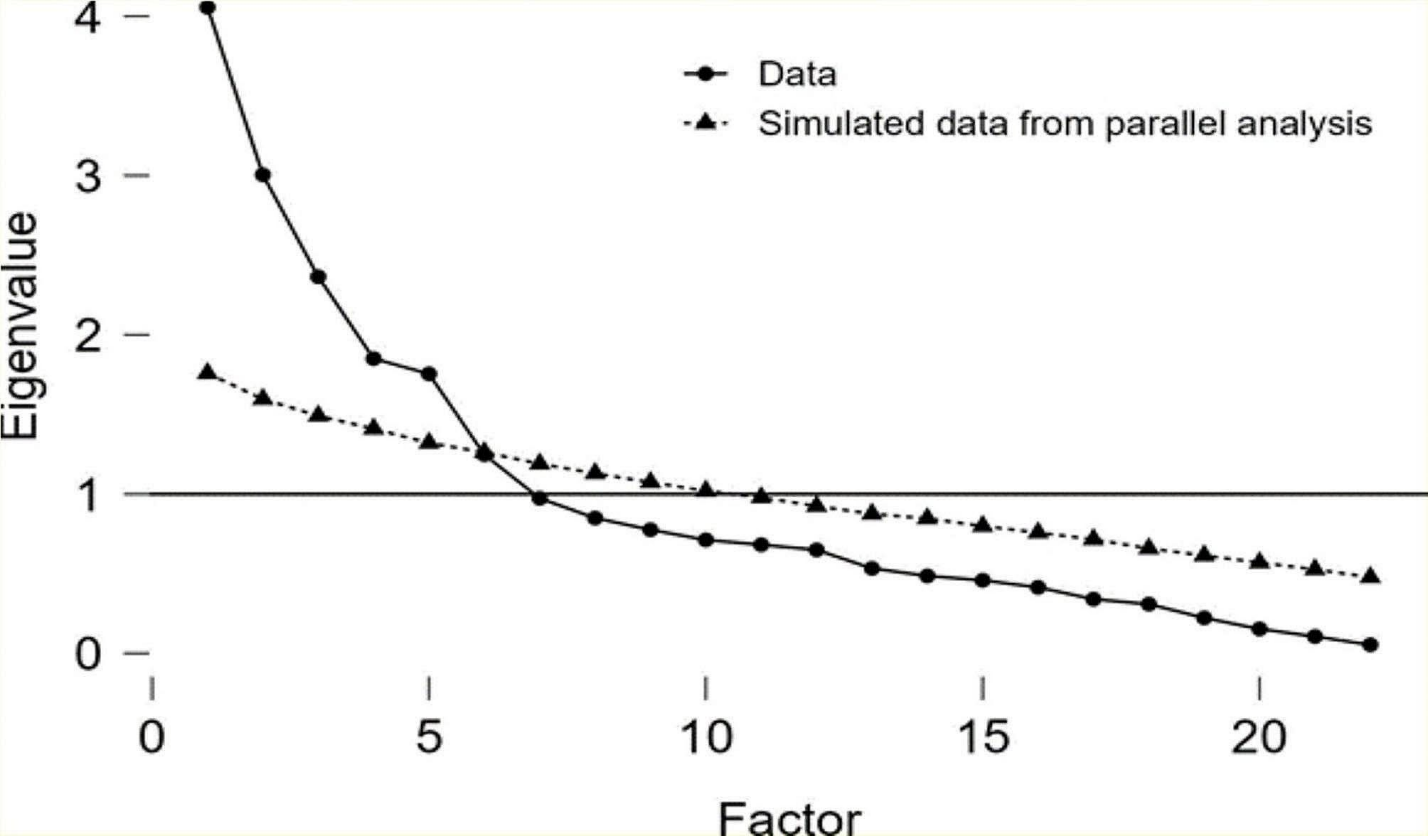
Factor load scree plot of the items for determining the number of extracted factors of the Iranian version of BCPS

**Fig. 2:**
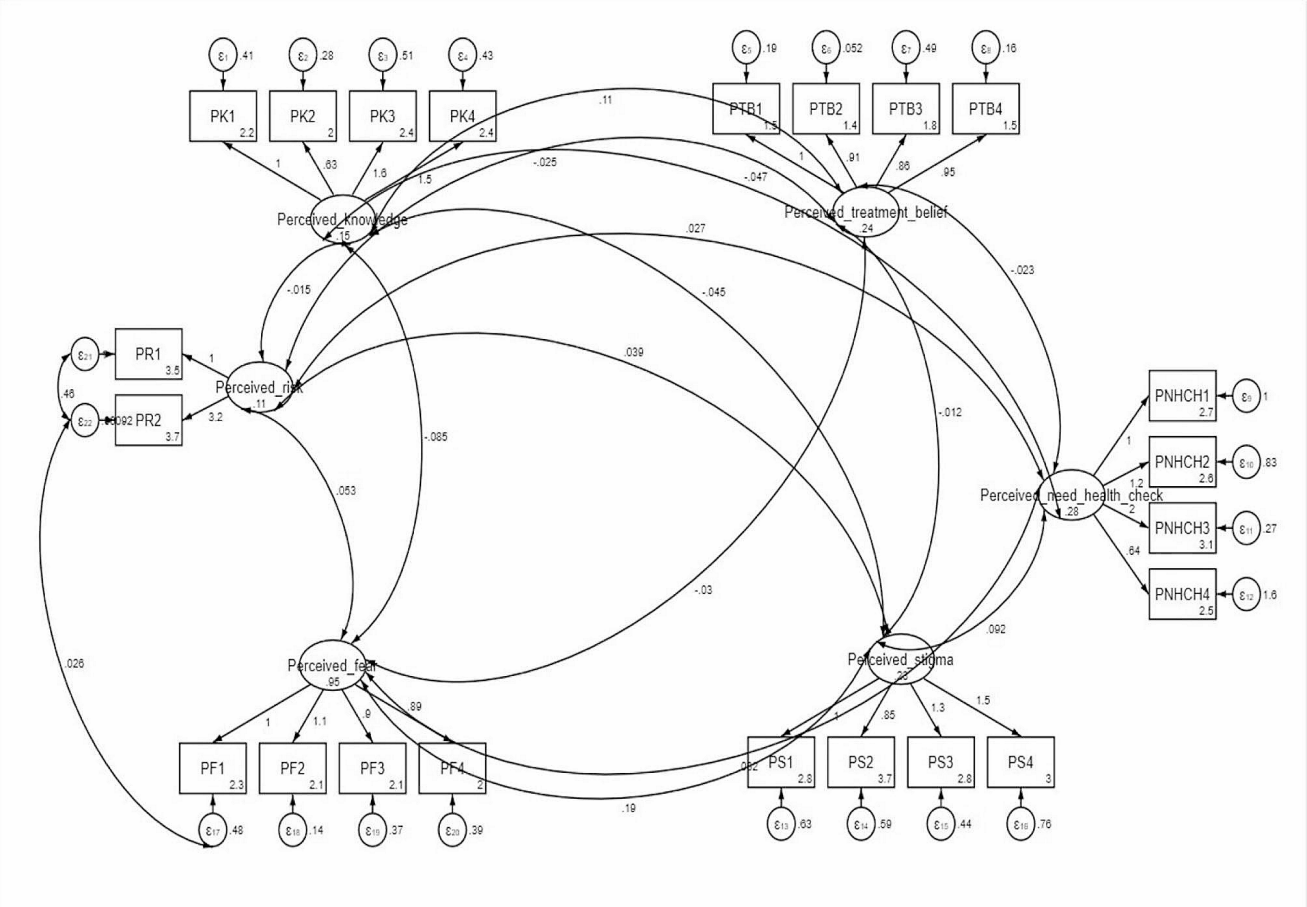
Factor structure model of the BCPS based on CFA. All factor-item relationships were significant (*P* < 0.05). Fc1: Perceived fear, Fc2: Perceived knowledge, Fc3: Perceived treatment belief, Fc4: Perceived risk, Fc5: Perceived need for health check, Fc6: Perceived stigma

The scree plot displayed the results of EFA, revealing six factors with eigenvalues > 1. These factors collectively accounted for 52% of the variance (Fig. 1). Table 3 presents the extracted components alongside the corresponding items associated with each factor. The initial factor examined in this study was Perceived fear, comprising a set of four questions that contributed to 13.60% of the overall variance. The second factor, referred to as Perceived knowledge, comprises a set of four questions that collectively account for 11.00% of the overall variance. The third and fourth factors were Perceived treatment belief and Perceived risk, respectively. These factors consisted of four and two questions, respectively, and accounted for 8.9% and 6.4% of the variance. The fifth factor, referred to as Perceived need for a health check, consists of four questions with a variance of 6.3%. Additionally, the sixth factor, known as “Perceived stigma,” comprises four questions that account for 5.8% of the total variance (Fig. 2). After preliminary psychometric testing, 24 items were factor-analysed providing a 22-item, six-factor scale. It is important to highlight that, in the original instrument, questions 9 and 23, respectively addressed the notions that “Breast cancer treatment does not change the outcome” and “The risk for breast cancer is higher in those with a family history of breast cancer,” were excluded from the EFA in the present study due to their factor loadings being less than 0.3. Consequently, there was a reduction in the number of instrument questions from 24 items in the original instrument to 22 items.

**Table 3:** Result of Factor analysis of the BCPS based on EFA (*n* = 172)

Scale item				Factors		
	1	2	3	4	5	6
**Factor 1: Perceived fear**						
1. It scares me to think of breast cancer	0.93					
2. I feel uncomfortable when I think of breast cancer	0.98					
3. It makes me feel uneasy to think about the breast cancer treatment process	0.96					
4. The thought of having breast cancer worries me	0.90					
**Factor 2: Perceived knowledge**						
5. My knowledge of breast cancer treatment is sufficient		0.70				
6. I think that I have sufficient knowledge of breast cancer		0.82				
7. I know what women who had breast cancer treatment should pay attention to		0.70				
8. I know how to be protected from breast cancer		0.55				
**Factor 3: Perceived treatment belief**						
9. It is important for early diagnosis and treatment to attend screenings regularly			0.54			
10. Early diagnosis of breast cancer increases the chances of recovery			0.78			
11. Breast cancer is a treatable disease			0.43			
12. Breast self-examination is important for early diagnosis and treatment			0.69			
**Factor 4: Perceived risk**						
13. I see myself under the risk for breast cancer				0.82		
14. I think that my chance of having breast cancer is high				0.77		
**Factor 5: Perceived need for health check**						
15. I do not go to the doctor unless there is a disease finding					0.59	
16. I forget to get a regular breast examination					0.66	
17. It does not come to my mind to go to a regular breast examination					0.71	
18. I am reluctant to be examined by a male doctor					0.39	
**Factor 6: Perceived stigma**						
19. Women with breast cancer experience problems in their sexual lives						0.72
20. Women with breast cancer cannot take care of their children						0.77
21. Women with breast cancer experience problems in their marriages						0.44
22. Breast cancer treatment makes a woman less beautiful						0.35
**% of variance observed**	13.60	11.00	8.90	6.40	6.30	5.80
**Total score**	52.00					

CFA was employed to examine the six factors that were derived from EFA. The findings indicate that the model has attained a level of fit that is considered optimal, thereby providing support for confirming the factor structure. The indicator $$\raisebox{1ex}{${x}^{2}$}\!\left/ \!\raisebox{-1ex}{$df$}\right.$$is found to be 2.029 (χ2 = 393.781, df = 194, P-value < 0.001). Additionally, the fit indexes TLI, CFI, NFI, and RFI all exceed the threshold of 0.9. Furthermore, the RMSEA and SRMR index values are both equal to 0.055, indicating a valid model.

### Hypothesis testing, responsiveness and interpretability

The hypothesis confirmation involved the computation of Spearman’s correlation coefficient between the BCPS and QLICP-BR V2.0, and the resulting coefficient of 0.55 indicated a statistically significant correlation. To assess the feasibility of the tool, the ceiling effect in the overall score of BCPS was found to be 0.3%. In the sub-domains, the ceiling effects for Perceived fear, Perceived knowledge, Perceived treatment belief, Perceived risk, Perceived need for a health check, and Perceived stigma were determined to be 2.2%, 0.4%, 0.3%, 1.1%, 0.3%, and 0.8%, respectively. The floor effect in the overall score of BCPS was observed to be 0.5%, while in the specific subdomains, it was found to be 26.9%, 3.5%, 3.2%, 1.3%, 4.6%, and 0.8%, respectively. It is noteworthy to mention that the MIC refers to a specific threshold value that delineates the smallest alteration in the measured parameter that holds clinical or practical significance. In this particular instance, the MIC was determined to be 3.92 units. Specifically, the study reveals that the MIC value surpasses the SDC value by 3.73 units. This observation indicates that the Iranian version of the measurement tool is sufficiently responsive. Put simply, the measurement tool can accurately identify and assess changes that hold significance or relevance within the given measurement framework. SEM of this study’s findings generally implies that the measuring device used exhibits a satisfactory level of precision. The comparison between the SDC and the MIC values further demonstrates the instrument’s capacity to consistently identify significant variations in the measured variable (Table 2).

## Discussion

The aim of this study was to assess the measurment properties of the Breast Cancer Perception Scale (BCPS) in Iranian women, according to the COSMIN checklist for the first time. The findings of the research substantiate the validity, reliability, responsiveness, and interpretability of the BCPS, which is grounded in the health belief model (HBM) when applied to Iranian women.

The HBM has been widely employed in the study of breast cancer diagnostic behaviors for an extended period [25–27]. The BCPS is a novel screening tool for breast cancer that has been developed utilizing the HBM [23]. Despite the presence of various tools in this domain, such as “belief in mammography” and “breast self-examination,” “perceived sensitivity toward breast cancer,” “perceived benefits and obstacles of mammography usage,” “fear of breast cancer (FBC)”, and “fatalism regarding cancer “, these instruments appear to lack practicality as they assess factors individually and fail to encompass all relevant domains. The BCPS demonstrates utility in its comprehensive coverage of various domains, particularly in assessing previously unmeasured aspects such as perceived knowledge, and mental measurements, including the perceived need for a health check, perceived stigma, perceived fear, and perceived risk [23].

Breast cancer perception is one of the most important indicators for preventing breast cancer and adopting protective behaviors against breast cancer. Proper perception of breast cancer serves as a motivator for women to adhere to breast cancer prevention methods. Despite the existence of various preventive and diagnostic methods for breast cancer, none of these methods will be effective until there is a proper perception of breast cancer. Therefore, the BCPS scale, considering important dimensions such as Perceived knowledge, Perceived treatment belief, Perceived need for health check, Perceived stigma, Perceived fear and Perceived risk, can play an important role in creating preventive behaviors against breast cancer [23].

In the current investigation, EFA was conducted on a set of 24 items of the instrument. The analysis yielded six factors, namely Perceived fear, Perceived knowledge, Perceived treatment belief, Perceived risk, Perceived need for a health check, and Perceived stigma. These factors aligned with the original instrument and collectively accounted for approximately 52% of the variance, while in the original instrument, they accounted for 74.36% of the variance [23]. To assess the validity of the instrument, the KMO measure was computed, yielding a value of 0.71. Additionally, the adequacy of the model was verified through Bartlett’s test, which yielded a significance level of 0.77 in the original study [23]. Furthermore, the reliability of the instrument was obtained ranging from 0.64 to 0.94 by using Cronbach’s alpha, and these values align with the original study’s reported range of 0.81 to 0.95 [23].

In the present study, the initial factor extracted during the exploratory EFA was identified as perceived fear. The influence of perceived fear on women’s adoption of protective behaviors against breast cancer can be observed. The findings of various studies indicate that a significant majority of women encounter fear regarding the potential diagnosis of breast cancer and the subsequent possibility of undergoing a mastectomy, either unilaterally or bilaterally, at some point in their lives [44, 45]. In a similar vein, a separate study indicated that women who exhibited a heightened FBC were found to undergo mammograms less frequently within a one-year timeframe in comparison to their counterparts [46]. In the study conducted by Aguirre et al., it was observed that young Spanish women exhibited a notable level of fear towards breast cancer, despite not expressing a general sense of concern regarding the disease. According to the study [47], it was found that 25.3% of the participants reported above-average FBC, while 59.7% reported high FBC. This finding implies that breast cancer may elicit a particularly strong sense of fear, even among young women who do not have significant health issues and have a low objective risk. This observation aligns with the findings of previous research [48, 49]. Furthermore, when comparing the findings of this study to previous research conducted within the past two decades, it becomes evident that the extent of fear induced by breast cancer has remained relatively stable despite favorable epidemiological advancements such as reduced mortality rates and enhanced treatment options [50].

The second factor that was extracted in this study related to perceived knowledge. Perceived knowledge encompasses biases, such as unrealistic optimism and implicit confidence [51]. The concept of perceived knowledge pertains to an individual’s level of knowledge and is not directly associated with one’s knowledge, specifically regarding breast cancer. The WHO has advocated for the adoption of breast cancer knowledge and awareness as a viable medical strategy for the management of breast cancer. This approach is deemed essential and should be universally implemented, irrespective of financial constraints. In this regard, Izanloo et al. demonstrated that a significant majority of the participants, totaling over 84%, exhibited a lack of knowledge regarding breast cancer and screening tests among 14- to 84-year-old Iranian women. The primary factors cited by women as barriers to undergoing screening tests were the absence of discernible symptoms or issues and their perception of the test’s necessity. A significant difference was observed in the level of women’s knowledge of breast cancer and screening tests concerning factors such as employment status, education level, and family history of breast cancer. However, no significant difference was found in the level of knowledge among women based on their marital status or income level [52]. Moreover, a study conducted by Mehejabin et al. sought to examine the level of knowledge regarding various aspects of breast cancer among women in Bangladesh. The findings revealed that a majority of the participants, exceeding 50%, possessed a limited understanding of the risk factors associated with breast cancer, indicating a significant lack of knowledge [53].

Perceived treatment beliefs constituted an additional factor. Perceived belief in treatment can be influenced by various factors, including women’s spiritual and religious beliefs, familial history of breast cancer treatment, and prior experiences with breast cancer treatment [45]. Concerning this matter, individuals’ perceptions of their treatment beliefs have the potential to influence their engagement in protective behaviors. The findings of the study conducted by Mehejabin et al. indicate that a considerable proportion of women hold the belief that breast cancer can be detected at a young age. Furthermore, the participants held the belief that early diagnosis of the disease could lead to its potential cure [53]. The aforementioned findings align with the results obtained from a research study carried out at Dhaka Medical College Hospital in Bangladesh, wherein 51.43% of female participants indicated that early detection of breast cancer leads to a potential cure [54]. Suwankhong and Liamputtong have posited that religious belief significantly influences individuals’ decision-making processes concerning treatment options and risk factors associated with breast cancer [55]. Yew et al., found a significant difference in the perceptions of breast cancer risk with religious affiliations, specifically between the Muslim and Buddhist cohorts. The impact of Islam and Buddhism on individuals’ lifestyles and health-related behaviors has been significant. Muslim women exhibited a profound conviction in the authority of God (Allah), whereas Buddhist women commonly invoked their karma [56].

Perceived risk, identified as an additional extracted factor, holds a significant influence over breast cancer protective behavior [45]. Observing the challenges and distress experienced by our beloved individuals throughout breast cancer treatment amplifies both the perceived fear and the perceived risk of breast cancer [57]. The primary determinant of health behaviors for breast cancer prevention, diagnosis, and control is the perceived risk. Conversely, establishing concordance between the perceived risk and the objective risk of developing breast cancer results in a more accurate and actual perception of the risk. Consequently, it can serve as motivation for fostering suitable health behaviors [58]. Hajian et al. [59], examined the perceived risk of breast cancer among 800 Iranian women about the actual risk. The findings of the study revealed that both women with a low and high risk of breast cancer exhibited a significantly higher perceived risk of the disease compared to their actual risk. This finding suggests a significant inclination towards pessimism in the assessment of breast cancer risk, consistent with previous research conducted in this domain [60, 61].

The results of this study also identified the perceived need for a health check as an additional extracted factor. One of the main obstacles to breast cancer screening among women is a diminished perception of the necessity for health screening. Women typically do not perceive the necessity of seeking medical attention unless they possess knowledge regarding the specific symptoms associated with a particular ailment [62]. Research findings indicate that women residing in developing nations often tend to decline the notion of early diagnosis and screening for breast cancer, primarily influenced by their cultural and personal beliefs. The aforementioned factor has a detrimental impact on the implementation of preventive measures aimed at mitigating the risk of breast cancer [63]. Therefore, variations in the perceived need for health screening can potentially impact individuals’ engagement in breast cancer protective behaviors. The scoping review study conducted by Omidi et al. examined the current status of breast cancer screening strategies and indicators among Iranian women. The study findings revealed that the prevalence rates of screening methods, including BSE, CBE, and mammography, among Iranian women were reported as 0-79.4%, 4.1–41.1%, and 1.3–45%, respectively [64].

Based on the HBM theory, Darvishpour et al. [25] posited that the decision of women to engage in breast cancer screening is influenced by factors such as self-efficacy and perceived benefits. Conversely, the presence of perceived barriers diminishes the likelihood of self-examination. According to Khazir et al., individuals who perceive fewer barriers are more likely to engage in breast cancer screening programs [65]. Abdel-Aziz et al. conducted a study utilizing EFA to examine the perceived barriers faced by women with breast cancer. Their findings indicate that personal fears, specifically fear of doctors/examiners, fear of screening results, and fear of the hospital environment, are the primary obstacles preventing women from utilizing free screening. These fears were identified as the main barriers based on their eigenvalue values, which exceeded 3.335, representing 30.4% of the barriers identified [66].

The final factor that was extracted pertains to the concept of perceived stigma. The symbolic significance of breasts for women stems from their association with childbirth, breastfeeding, childrearing, and sexual desires. Consequently, the symbolic significance associated with this phenomenon may impede women from accessing necessary healthcare services, interventions, or diagnostic procedures [67]. Furthermore, the absence of discussion regarding breast cancer and screening behaviors may be associated with societal stigmatization and cultural taboos surrounding the topic of breasts [68]. It is well known that stigma plays a significant role in the psychological distress that breast cancer-diagnosed women experience. The occurrence of rejection, blame, or devaluation is what defines the social phenomenon known as stigma. This arises from the personal experience, perception, or rational expectation of an unfavorable social evaluation directed toward an individual or a collective entity [69]. It was found that around 76.7% and 8.7% of breast cancer survivors reported moderate and high levels of stigma, respectively [70]. Based on prior research, it has been established that the perceived stigma among individuals diagnosed with breast cancer has significant adverse consequences for their overall well-being and health-related outcomes. These repercussions encompass various aspects such as sexual dysfunction, depressive symptoms, compromised sleep quality, reduced inclination to seek medical assistance, and diminished quality of life [71].

In terms of clinical application, the use of this scale is considered to save time and enable early detection of breast cancer during assessment. Utilizing this screeninig tool by health care providers improves a quick and comprehensive attitude toward breast cancer perception among women. The main advantage of BCPS is that it helps more subjective measurements compared to other scales in this area. In addition, its goal is to evaluate the relationship between breast cancer and diagnostic behaviors for breast cancer (such as maintaining healthy behaviors like diet, physical activity, mammography, breast self-examination, and clinical breast examination), knowledge about breast cancer, and family history of breast cancer [23].

### Strength and limitation

The present study possesses several notable strengths. Firstly, it is the first study to assess BCPS among Iranian women. Secondly, the study adheres to the COSMIN checklist, ensuring methodological rigor. Additionally, the study incorporates both the DP and FB methods for the translation process, effectively addressing the limitations associated with the FB method. Lastly, the study includes a comparative analysis of BCPS with other versions.

However, it is important to acknowledge the limitations of the current study. These limitations include the lack of criterion validity calculations due to the lack of a gold standard, the lack of an assessment of cross-cultural validity and the possibility of bias from participants’ tendency to give socially desirable answers when using self-reported measures. As we conducted this study in Tabriz-Iran, should be cautious about the generalizability of findings. In conclusion, it is recommended that future research endeavors employ a larger sample sizeand assess the measurment properties in diverse contexts.

## Conclusions

The obtained findings suggest that the Iranian version of the BCPS demonstrates satisfactory measurment properties for assessing the perception of breast cancer among Iranian women. Furthermore, it exhibits favorable responsiveness to clinical variations. The assessment of women’s perceptions of breast cancer is imperative for the advancement of preventive behaviors against this disease. The present scale can be employed for the assessment of the association between breast cancer and behaviors related to breast cancer diagnosis, including breast self-examination, clinical breast examination, mammography, and the adoption of healthy behaviors such as diet and exercise. Finally, it can be utilized to investigate the correlation between breast cancer knowledge and family history.

## Data Availability

The datasets generated and/or analyzed during the current study are not publicly available due to the limitations of ethical approval involving the patient data and anonymity, but are available from the corresponding author upon reasonable requests.

## Abbreviations

BCPS: Breast Cancer Perception Scale
COSMIN: Consensus-Based Standards for the Selection of Health Status Measurement Instruments
BC: Breast Cancer
GLOBOCAN: Global Cancer Incidence Mortality and Prevalence
WHO: World health organization
EMR: Eastern Mediterranean Region
ASR: Age-standardized rate
HRT: Hormone replacement therapy
VAS: Visual Analouge Scale
HBM: Health Belief Model
BSE: Breast self-examination
CBE: Clinical Breast Examination
HR-PROs: Health related-patient reported outcomes
DP: Dual pannel
FB: Forward-backward
EFA: Exploratory factor analysis
CFA: Confirmatory factor analysis
ICC: Intra-class Correlation Coefficient
SD: standard deviation
CVI: Content validity index
CVR: Content validity ratio
SEM: Standard error of measurment
SDC: Smallest detectable change
MIC: Minimal important change
df: Degree of freedom
AVE: Average variance extracted
KMO: Kaiser-Meyer-Olkin
RMSEA: Root mean squared error of approximation
CFI: Comparative fit index
TLI: Tucker–Lewis index
